# A New Cycle Slip Detection and Repair Method Using a Single Receiver’s Single Station B1 and L1 Frequencies in Ground-Based Positioning Systems

**DOI:** 10.3390/s20020346

**Published:** 2020-01-08

**Authors:** Xinyang Zhao, Zun Niu, Gaoxu Li, Qiangqiang Shuai, Bocheng Zhu

**Affiliations:** Department of Electronics, Peking University, Beijing 100871, China; 1801111306@pku.edu.cn (X.Z.); 1601111239@pku.edu.cn (Z.N.); ligaoxu@pku.edu.cn (G.L.); shuaiqq@pku.edu.cn (Q.S.)

**Keywords:** cycle slip detection, ground-based navigation, multipath effect, Particle Swarm Optimization algorithm

## Abstract

The detection and repair of the cycle slip is a key step for high precision navigation and positioning in indoor environments. Different methods have been developed to detect and repair cycle slips for carrier phase processing. However, most approaches are designed to eliminate the effects of the ionosphere in an outdoor environment, and many of them use pseudorange (code) information that is no longer suitable for indoor multipath environments. In this paper, a method based on the geometry-free combination without the pseudorange data is proposed to detect and fix cycle slips. A ground-based navigation system is built for data collection. Unlike the traditional dual-frequency cycle slip detection method, the Beidou B1, GPS L1 carrier phase combination is used instead of the B1, B2, or L1, L2 carrier phase combination, Ublox is used for data collecting. For fixing the cycle slips quickly, an improved adaptive Particle Swarm Optimization (PSO) algorithm is employed. We compared the performance of the new method with the existing two methods using simulated data in different conditions. The results show that the proposed method has better performance than other methods.

## 1. Introduction

The past decade has seen the rapid development of Ground-based positioning systems; it has the potential to deliver sub-centimeter positioning precision indoors using a low-cost receiver [1]. By using the GNSS-like signals, Ground-based positioning systems can provide stand-alone positioning services and improve the positioning ability of GNSS, becoming an indispensable part of navigation [2]. Ambiguity resolution is a key issue for indoor precise point positioning. However, the occurrence of cycle slips frequently in carrier phase measurements due to multi-path effects in an indoor environment is one issue constantly encountered in achieving high precision. When an unknown integer cycle slip occurs, the integer ambiguity will be biased. Even if the slip is only one cycle, the resultant range error is 19 cm (GPS L1 signal). Therefore, accurately detecting and repairing the cycle slips is an important pre-processing step in high precision indoor positioning.

Significant effort has been devoted to cycle slip detection and repair algorithms over the past decades. Most of the cycle slip detection methods have been proposed for GNSS in an outdoor positioning environment, but many of these existing methods have their shortcomings for indoor cycle slips detection and repair. For example, some studies employed Bayesian detection [3] to improve cycle slip detection and correction. However, the accuracy of Doppler integration is limited to low frequency. The complexity of the high-order differences method [4] is lower and easily implemented, but the detection accuracy becomes unpredictable in the case of the low sampling rate. The polynomial fitting method is robust when the signal has discontinuities. However, the performance is poor if the sampling interval is larger than 1 s [5]. The Melbourne–Wubbena (M–W) linear combination [6,7] has been widely used in cycle slip detection in double-differenced as well as undifferenced observations. The major advantage of M–W combination is that it is not only ionosphere-free but also geometry-free. However, multi-path effects are severe in ground-based positioning. The error of the code measurement could be relatively large, resulting in a low-accuracy initial cycle slips detection by the M–W combination. A new approach using a single GPS receiver’s dual-frequency data has been proposed by [8]. However, this method is only suitable for short sampling periods. Liu [9] modified this approach by introducing the ionospheric total electron contents rate (TECR) together with the time-differenced (TD) Hatch–Melbourne–Wübbena (HMW) linear combination. Cai [10] introduced an approach that uses a forward and backward moving window to determine the cycle slips. Both of them can reduce the noise of the code measurement. However, compared to GNSS, the signals of ground-based navigation systems are unaffected by the ionosphere. In this case, the mathematical models of these existing methods established by modifying the ionospheric parameters are no longer applicable.

Studies over the past decade have shown the advantages of the combination of B1 with B2 [11] and L1 with L2 [12,13] for cycle slip detection. However, few studies have investigated the performance of the combination of B1 (1561.098 MHz) and L1 (1575.42 MHz), and much uncertainty exists, such as whether Ublox can receive signals from the same pseudolite GPS and Beidou at the same time, and if it can be used as a promotion of ground-based pseudolite systems since their frequency difference is 14.3 MHz, smaller than 354 MHz (B1 B2) and 347.8 MHz (L1 L2).

We present a new ground-based cycle slip detection and repair method based on carrier phase combination of B1 and L1 frequencies from a single station. Many cycle slip detection and repair methods use the phase/code range combinations. To reduce the impact of multipath, the approach proposed in this paper utilizes only phase observation, and thus, can suitably be implemented in the ground-based positioning systems. The method has resulted in a further reduction in observation noise levels in an indoor environment and allows a long-time sampling interval. The numerical results indicate that the new algorithms can effectively detect and repair cycle slips in multipath environment conditions. By using an adaptive weight Particle Swarm Optimization (APSO) algorithm, this new method can quickly find the cycle slip candidates without the search space by code range. The latter capability contributes to the development of new algorithms for indoor navigation cycle slip repair.

In the following sections, the new ground-based cycle slip detection model is established. Subsequently, an enhanced new algorithm based on the PSO is proposed to repair the cycle slips, and the dual-frequency observation of carrier phase measurement is introduced. Besides, a real-world experiment is conducted to collect data and demonstrate the proposed algorithm with an in-house-developed prototype ground-based navigation system. Then, numerical results are presented to prove the effectiveness of the new method. Conclusions and future work are summarized in the last section.

## 2. Adaptive Particle Swarm Optimization (PSO) Algorithm Mathematical Model

### 2.1. Function Model

A typical ground-based positioning system that includes more than four pseudo satellites comprises base stations and user receivers. The base stations share a common clock source through a wired connection to keep time synchronization. Receivers obtain the signals broadcast by base stations and then process the signals for getting code and phase measurements. Subsequently, the receiver clock error and user position are solved by the phase observation equations. Although the dual-frequency receiver carrier phase and code observations have many errors, the stochastic noises and multi-path are independent. A combination of different coefficient values (k1 k2) generates different characteristic measurements. An important procedure for the linear combination of dual-frequency measurements is to filter through all valid combinations to make the corresponding combined measurement values ionosphere-free or geometry-free, which is beneficial to solve the ambiguity and improve the relative positioning accuracy. Here, we use geometric models. Firstly, there is no ionospheric delay term in the observation equation, since the signal of ground-based navigation does not pass through the ionosphere [1,14]. This makes the approach more reasonable and robust. Secondly, the non-dispersive errors and the geometry term q can be fully eliminated by a geometry-free phase combination. The geometry-free combination reads [15]:

(1) $$\lambda_{B1}\Delta \Phi_{B1}-\lambda_{L1}\Delta \Phi_{L1}=\lambda_{B1}\Delta N_{B1}-\lambda_{L1}\Delta N_{L1}+\lambda_{B1}\Delta e_{B1}+\lambda_{L1}\Delta e_{L1}+\Delta M_{B1}+\Delta M_{L1}$$

**Figure 1:**
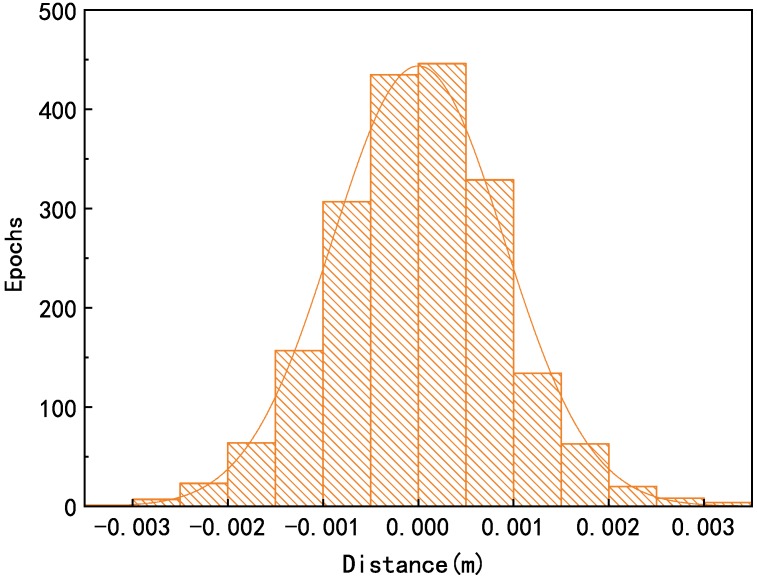
Normal distribution due to multipath and noise.

$\lambda_{B1}$ and $\lambda_{L1}$ are the wavelength of the corresponding Beidou B1 and GPS L1 signal, respectively. The Φ is the received carrier phase observable value in units of cycles; $\Delta \Phi_{B1}$ and $\Delta \Phi_{L1}$ are the number of changed values for B1 and L1, respectively. *N* is the integer phase ambiguity in units of cycles, *M* stands for the multipath error in units of length. $\Delta M_{B1}$ and $\Delta M_{L1}$ representing distance changes of the two frequency due to the multipath effect, the carrier phase measurements bring in much lower multipath error than thermal noise and code data. e represents the carrier phase thermal noise; Note that there is no ionospheric delay term in the ground-based navigation, and the multipath errors are difficult to model explicitly. We can combine the effects of multipath and Gaussian distribution into an extended Gaussian distribution. That means $\lambda_{B1}\Delta \Phi_{B1}-\lambda_{L1}\Delta \Phi_{L1}$$∼N\left(0,\sigma \right)$ when the ΔN=0. The statistical data proves that the model conforms to the normal distribution. We use the Shapiro–Wilk method to detect the normal distribution of the results of Equation (1). The *p*-value is 0.66, and the conclusion is at the 0.05 level, the data is significantly drawn from a normally distributed population, as shown in Figure 1.

For the satellite, we can assume that the carrier phase standard deviation of the two frequencies is the same. $\sigma_{B1}=\sigma_{L1}$ where $\sigma_{B1}$ and $\sigma_{L1}$ represent the standard deviation of B1L1 carrier phase thermal noise, $\sqrt{2}$ reflects the between-epoch differencing respectively.

(2) $$\sigma =\sqrt{2}\cdot \sqrt{\lambda_{B1}^{2}+\lambda_{L1}^{2}}\cdot \sigma_{B1}$$

The judgment of the occurrence of the cycle slip is as follows:

(3) $$\left|\lambda_{B1}\Delta \Phi_{B1}-\lambda_{L1}\Delta \Phi_{L1}\right|>f\cdot \sigma$$

*f* is a constant related to detection sensitivity, we set *f* to 3 in the examination. When cycle slip occurs, we use APSO to find the candidates and fix the cycle slip.

### 2.2. Parameters Setting of APSO Algorithm

Particle swarm optimization (PSO) is an evolutionary computation technique. It has drawn widespread attention in the last decades, and was developed by Kennedy and Eberhart [16,17]. There are two main reasons why we chose the PSO algorithm. One is that it has excellent global search capabilities, and the other is that it has good efficiency. The cycle slip repair process is to find $\Delta N_{L1}$ and $\Delta N_{B1}$ in Formula (4). In other words, removing the cycle slip candidates from the carrier phase raw data should make the repaired carrier phase data most possibly pass the cycle slip detection (Formula (3)).

(4) $$fitness={\left(\lambda_{L1}\left(\Delta \Phi_{L1}-\Delta N_{L1}\right)-\lambda_{B1}\left(\Delta \Phi_{B1}-\Delta N_{B1}\right)\right)}^{2}\to min$$

In indoor environments where multipath effects are severe, we no longer use pseudo-range information, which makes the cycle search space unpredictable. In order to ensure the accuracy of cycle slip repair, when using traditional algorithms, we need to traverse the candidates one by one in a large search space, which requires a large amount of calculation. However, we do not need to specify the search space when using the APSO algorithm. The initialization is a random number. Using the APSO algorithm, we can quickly find candidates, even if the number of cycle slips is a few hundred. Fast convergence and no need to define a search space make the APSO algorithm suitable for cycle slip detection in indoor environments. The position and velocity of the particle i in the N-dimensional space are expressed as a vector. First, determine the initial position and velocity of the particle in the feasible solution space and velocity space. The basic parameters of the particle swarm are as follows:

Particle swarm is a population of n particles: $X=(X_{1},X_{2},X_{3},X_{4},X_{5},\ldots ,X_{n})$
The particle *i* represents a two-dimensional vector: $X_{i}={\left(x_{i1},x_{i2},x_{i3},x_{i4},x_{i5},\ldots ,x_{iD}\right)}^{T}$$\Delta N_{L1}$ and $\Delta N_{B1}$ are a two-dimensional vector in the APSO algorithm, and represented by $x_{i1},x_{i2}$.
The speed of the particle *i* is: $V_{i}={\left(V_{i1},V_{i2},V_{i3},V_{i4},V_{i5},\ldots ,V_{iD}\right)}^{T}$
The local extremum value is: $P_{i}={\left(P_{i1},P_{i2},P_{i3},P_{i4},P_{i5},\ldots ,P_{iD}\right)}^{T}$
The global extremum value is: $P_{g}={\left(P_{g1},P_{g2},P_{g3},P_{g4},P_{g5},\ldots ,P_{gD}\right)}^{T}$

The speed update expression is:

(5) $$V_{id}^{k+1}=V_{id}^{k+1}|+c_{1}r_{1}\left(P_{id}^{k}-X_{id}^{k}\right)+c_{2}r_{2}\left(P_{pd}^{k}-X_{id}^{k}\right)$$

$c_{1}$ and $c_{2}$ are the parameter values, $r_{1}$ and $r_{2}$ are two random values. The location update expression is:

(6) $$X_{id}^{k+1}=X_{id}^{k}+V_{id}^{k+1}$$

In each iteration, the results of the objective function (fitness) of all the particles are evaluated to determine the best position Pbest that each particle has reached at time *t*, and the best position gbest found by the group. Update the speed and position of each particle by tracking these two best positions.

(7) $$v_{i,j}\left(t+1\right)=v_{i,j}\left(t\right)+c_{1}r_{1}\left[p_{i,j}-x_{i,j}\left(t\right)\right]+c_{2}r_{2}\left[p_{g,j}-x_{i,j}\left(t\right)\right]$$

(8) $$x_{i,j}\left(t+1\right)=x_{i,j}\left(t\right)+v_{i,j}\left(t+1\right),j=1,\ldots ,d$$

$c_{1}$ and $c_{2}$ are positive learning factors or called acceleration constants. $r_{1}$ and $r_{2}$ are the evenly distributed random numbers between 0 and 1. The speed can be limited by setting the speed interval of the particles.The speed update expression as follows:

(9) $$v_{i,j}\left(t+1\right)=v_{i,j}\left(t\right)+c_{1}r_{1}\left[p_{i,j}-x_{i,j}\left(t\right)\right]+c_{2}r_{2}\left[p_{1,j}-x_{i,j}\left(t\right)\right]$$

In order to control the flying speed of particles effectively, Shi and Eberhart [18] introduced the inertia weight coefficient *w* into the algorithm model to realize the effective control and adjustment of the flying speed of particles. The mathematical expressions for the velocity and position of particles correspondingly change to:

(10) $$v_{i,j}\left(t+1\right)=wv_{i,j}\left(t\right)+c_{1}r_{1}\left[p_{i,j}-x_{i,j}\left(t\right)\right]+c_{2}r_{2}\left[p_{g,j}-x_{i,j}\left(t\right)\right]$$

(11) $$x_{i,j}\left(t+1\right)=x_{i,j}\left(t\right)+v_{i,j}\left(t+1\right),j=1,\ldots ,d$$

It can be seen from the above formula that as the flying speed of the particles increases with a larger *w*, the particles will search globally with wide steps. Particle step size decreases with a small *w*, and local search ability becomes strong. $wv_{i,j}\left(t\right)$ can be regarded as the previous velocity or inertia of the particles. $c_{1}r_{1}\left[p_{i,j}-x_{i,j}\left(t\right)\right]$ can be regarded as the cognitive behavior of particles. $c_{2}r_{2}\left[p_{g,j}-x_{i,j}\left(t\right)\right]$ can be considered as the social behavior of particle. $x_{i,j}\left(t+1\right)$ shows the positional adjustment of the particles due to mutual influence in the solution space. In the whole solution process, inertia weight w, acceleration factor $r_{1}$ and $r_{2}$, and maximum speed Vmax, jointly maintain the balance of particles on global and local search capabilities.

In order to find the cycle slip candidates, we use the adaptive weight PSO algorithm, which is suitable for *B*1 *L*1 dual-frequency cycle slip repair. To balance the global search ability and local improvement ability of the PSO algorithm, the expression becomes as follows:

(12) $$w=w_{min}-\frac{\left(w_{max}-w_{min}\right)*\left(f-f_{min}\right)}{f_{avg}-f_{min}},f\leq f_{avg}$$

(13) $$w=w_{max},f>f_{avg}$$

where $w_{max}$ and $w_{min}$ represents the maximum and minimum values of *w*, respectively, and f represents the current objective function (fitness) value of the particle. $f_{avg}$ and $f_{min}$ represent the average target value and the minimum target value of all current particles, respectively. The inertia weight automatically changes and is called adaptive weight.

### 2.3. The Algorithm Flow of Cycle Slip Repair

The steps of using the adaptive weight PSO algorithm to repair the cycle slip are as follows:

1. Initialize the velocity and position of each particle in the population. In this experiment, each particle contains two unknowns of $\Delta N_{L1}$ and $\Delta N_{B1}$, and the dimension is two. According to the characteristics of cycle slip, we set the initial values of speed and position to random integers. We also set the current historical optimal position $P_{best}$ of each particle to the initial position, and take the optimal global position of the particle group as the optimal value in $g_{best}$.
2. Calculate the function fitness value of each particle. Store the best position and fitness of $\Delta N_{L1}$ and $\Delta N_{B1}$ and select the best particle position from the population as the location of the population.
3. Adjust the velocity and position of the particles according to the updated Equations (10) and (11) where parameter w is the adaptive weight value.
4. Calculate the fitness of each particle after the position updated, compare the fitness result of each particle with the fitness value corresponding to the best position $P_{best}$ that it has experienced before. If the result is better, use its current position as the $P_{best}$ of the particle.
5. Compare the fitness of each particle to the best position $g_{best}$. If this result is better than $g_{best}$, update $g_{best}$ with this value.
6. Check the termination condition (the maximum number of times is reached or the optimal solution stops changing). If the preset condition is not satisfied, return to step 3. If the preset condition is met, the iteration stops, the size of the cycle slips of $\Delta N_{L1}$ and $\Delta N_{B1}$ can be found.

### 2.4. The Traditional Doppler and the High-Order Time Difference Method

We compared the performance of the proposed cycle slip detection method with the Doppler algorithm and the higher-order time difference of carrier phase measurement method. The detection and repair process of the latter two methods was proposed in [15].

Doppler represents the instantaneous change rate of the carrier phase, which is a very robust measurement for cycle slip detection. The frequency of the received signal differs from the transmitted signal, which is caused by the relative motion of the receiver, and the transmitter is named the Doppler shift. The method predicts the growth in the carrier phase observations between adjacent epochs by using the observed Doppler shift. Ignoring the influence of noise, a deviation between the observed and predicted the carrier phase observation indicates a cycle slip. The size of the deviation also indicates the number of cycle slips. Therefore, Doppler measurement is an alternative way to detect and repair cycle slips. The relationship between the change of carrier phase in adjacent epochs and Doppler is as follows:

(14) $$\Delta \Phi_{Li}=-\left[D_{Li}\left(t\right)+D_{Li}\left(t-1\right)\right]\cdot dt/2$$

where $d_{t}$ represents the time interval between epochs, D is the measurements of Doppler frequency. By differencing the carrier phase measurements of adjacent epochs, we can obtain the change of carrier phase.

The high-order time difference is another commonly used approach for cycle slip detection and correction operating on carrier phase measurements of frequency. The subtraction can eliminate these errors between the epochs. This high-order time difference algorithm employs only the phase data, and it is less affected by the multipath in an indoor environment. The high-order method is based on the phase data of the previous epochs, and normally four or more observation epochs are needed. The phase data show a smoothed curve with time. When the frequency is low, the small noise will be amplified several times during the difference. However, a cycle slip is also amplified in high order differences, making the difference suddenly increase. The size of the cycle slips is left in the subtraction, and the sudden increase in value reflects the number of cycle slips that occurs and can be repaired later. As for the n-order time difference detection, there is n+1 known phase value, and the inner difference is the function value located discretionally between these n+1 values. We used a third-order high-order difference to process the carrier phase values of GPS and Beidou, respectively.

## 3. Experiments by Ground-Based Navigation System

**Figure 2:**
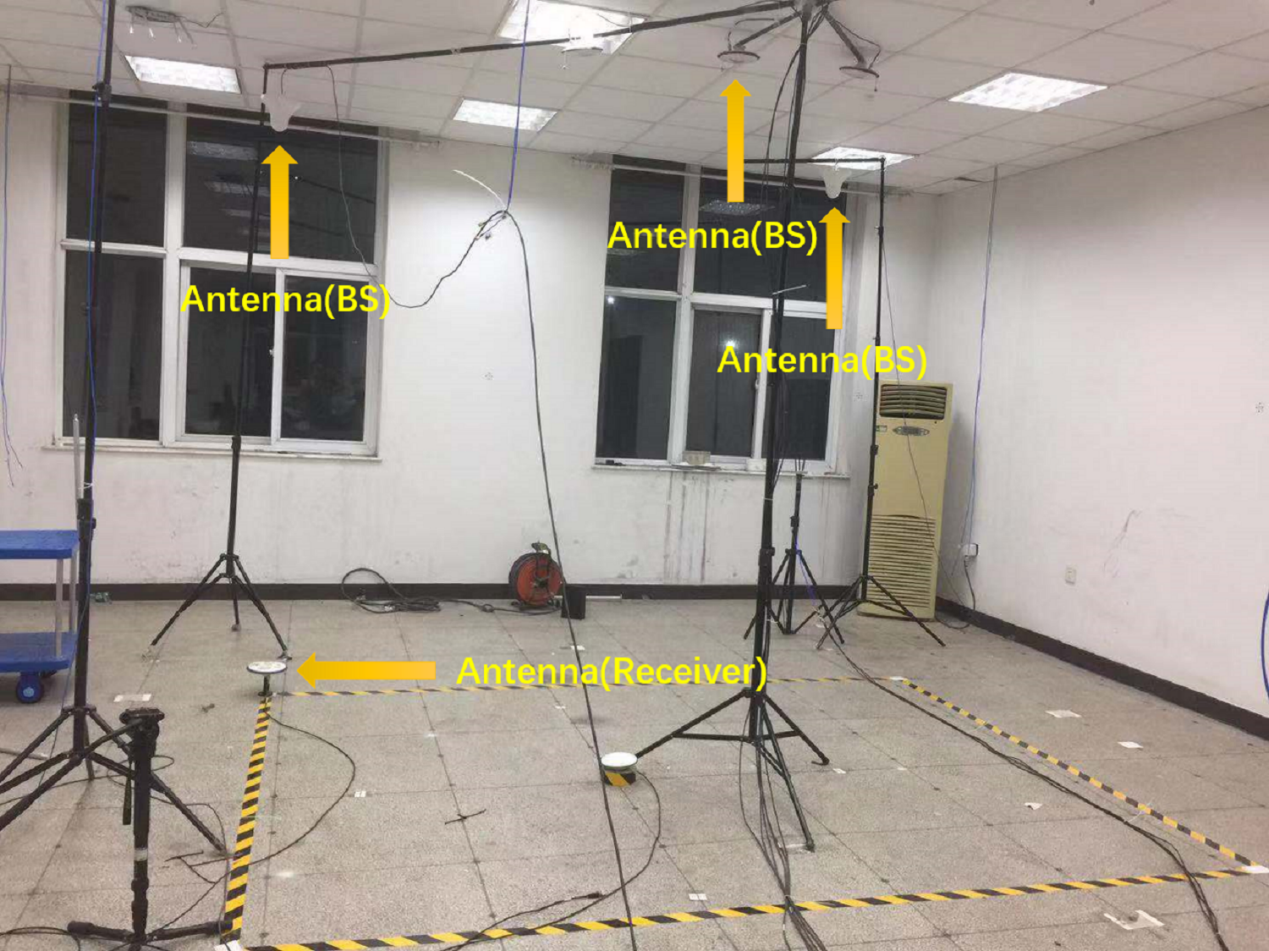
Ground-based navigation system.

The ground-based navigation system consists of seven base stations. This system is built in a 6 m by 10 m room, as shown in Figure 2. The signal transmission baseband unit of the station is a multi-channel RF module (ad9371), which is driven by the DSP and FPGA. And each station can transmit signals of two frequencies: Beidou B1, GPS L1. The base station comprises an antenna. All the transceivers shared a common clock source for synchronization.

In order to enable the receiver to receive the signals of all pseudolites while reducing the impact of multipath, we put the pseudolites in a high position and keep the distance between pseudolites to avoid signal interference. We use the Electronic Total Station for antennas coordinates determination, and the calibration accuracy is 1–2 mm. The coordinates of the pseudolite are shown in the Table 1.

**Figure 3:**
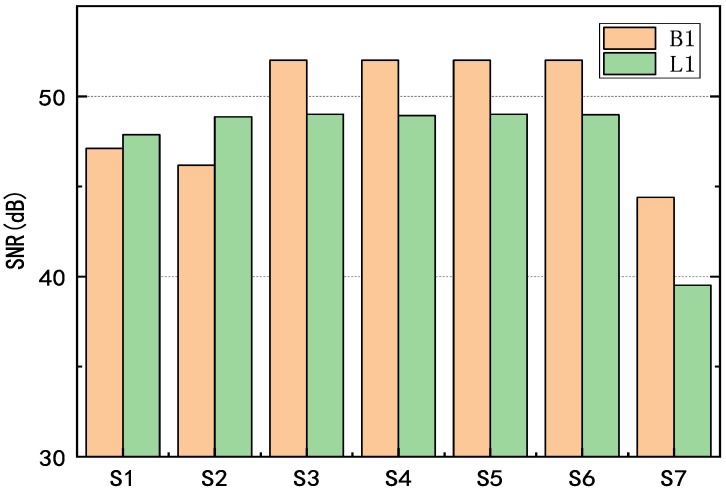
Average SNR (signal-to-noise ratio) values of the pseudolite.

A static test was performed at a known location (‘receiver’ in Figure 2), (−0.607, −3.403, 0.079), to assess the pseudolite signal quality and indoor positioning accuracy as well. The receiver consisted of an antenna and a Ublox module connected to the computer. Then, we used this receiver to collect data for cycle slip detection. We first measured the signal strength of each transmitter B1 and L1 frequency, respectively. The signal-to-noise ratio (SNR) values of the seven pseudolites are recorded. Since their signals were stable under static conditions, the signal strength fluctuations were within 4 dB. We collected SNR values of two thousand epochs and calculated their mean and standard deviation separately. We plot the average SNR of the B1 and L1 signals for each pseudolite in Figure 3. Moreover, we list the maximum and minimum SNR values and variances in Table 2. As can be seen from the figure, the signal strength from all the pseudolites was good; most of them exceed 45 dB, and the average signal strength of B1 is better than L1.

**Figure 4:**
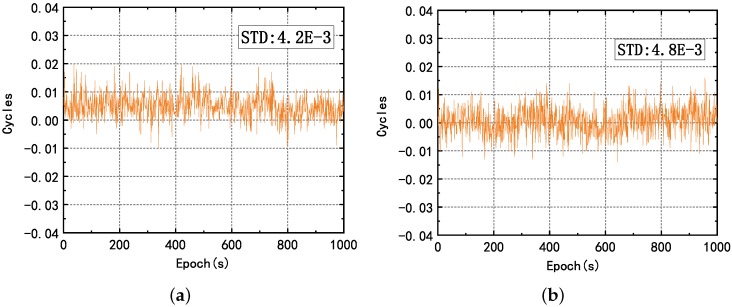

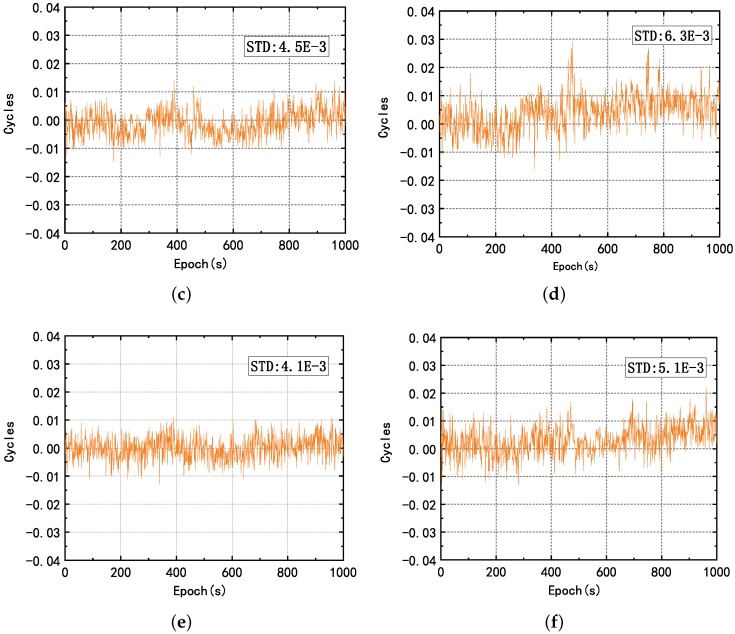
Single-difference carrier phase values between S1 and other pseudolites: (**a**) pseudolite 1 with pseudolite 2; (**b**) pseudolite 1 with pseudolite 3; (**c**) pseudolite 1 with pseudolite 4; (**d**) pseudolite 1 with pseudolite 5; (**e**) pseudolite 1 with pseudolite 6; (**f**) pseudolite 1 with pseudolite 7.

Another way to assess system quality, pseudolite synchronization, and the positioning accuracy achieved by the carrier-phase is to compute their carrier-phase single-difference measurements between the pseudolites proposed in [1]. We can directly resolve the ambiguity based on known coordinate information. Then, the single-differenced carrier phase value eliminates the clock error and shows errors due to the multipath. Without loss of generality, we use S1 as the Master pseudolite and then computed the single-difference carrier phase values between S1 and other pseudolites. The result of the difference is shown in Figure 4, and the value of the standard deviation is also marked in the figure. Except for B1 of S7, the standard deviation values are within $6\times 10^{-3}$ cycles, which means that the system is well synchronized and the clock skew is small. If we use the DKPI positioning method proposed by [19], the change in coordinates can be expressed by the change of the carrier phase difference. The small noise of the carrier phase difference (less than 0.01 cycles) represents the distance difference noise is less than 0.2 mm, indicating that the system can achieve centimeter-level positioning accuracy. Since the B1 frequency signal quality are higher than L1, the subsequent experimental analysis mainly focuses on B1.

## 4. Numerical Results and Discussion

**Figure 5:**
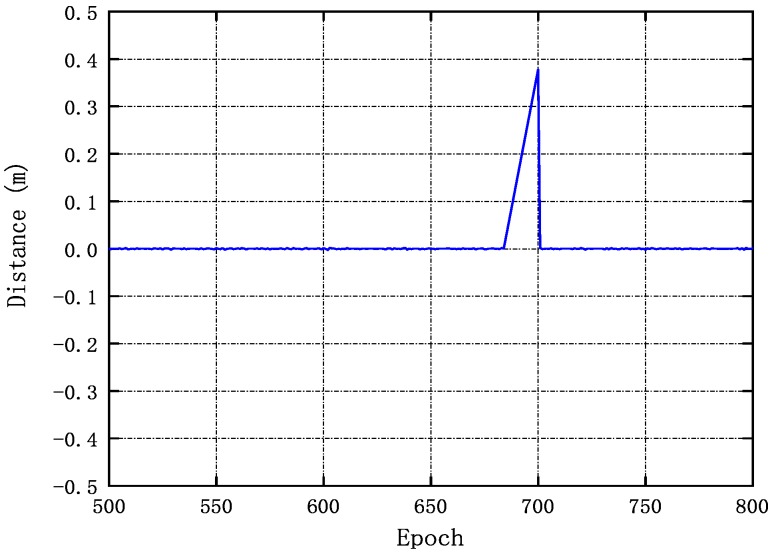
Real data with real cycle slips detection results obtained by the proposed indoor dual-frequency cycle slip method.

The authors conducted the experiment use Ublox with a one-second intervals. The GPS signal and the BDS signal are lost from the 684th epoch to the 700th epoch of the base station B7. Cycle slips were detected using the three methods. Then, we use the APSO algorithm to repair cycle slips. The same sizes for the slips have been obtained using the APSO algorithm and the Doppler method and the high-order difference method. We get $\Delta N_{L1}$ is 4 and $\Delta N_{B1}$ is 2. This result validates the practicality of the new method. We plot the test results using the proposed method as shown in Figure 5.

In order to measure the situation of cycle slip detection and repair comprehensively, we then use the simulated cycle slip for the collected carrier phase values. When cycle slip occurs, we use the PSO algorithm to repair it. Since the cycle slip generally occurs as an integer. In the process of searching for unknown cycle slips, we set the unknowns as integers. Therefore, the search range is an integer field.

We analyze the following situations proposed in [20]. (1) One or two cycle slips randomly assigned on B1 phase values. (2) Three to ten cycle slips randomly assigned on B1 phase values. (3) More than ten to dozens of cycle slips randomly assigned to B1 phase values of different epochs. (4) Random cycle slips assigned on both B1 and L1 carrier observations on different epochs. In order to investigate the performance of the method, the simulated results of S1 under different methods were plotted, and the sampling period was found to be one second.

**Figure 6:**
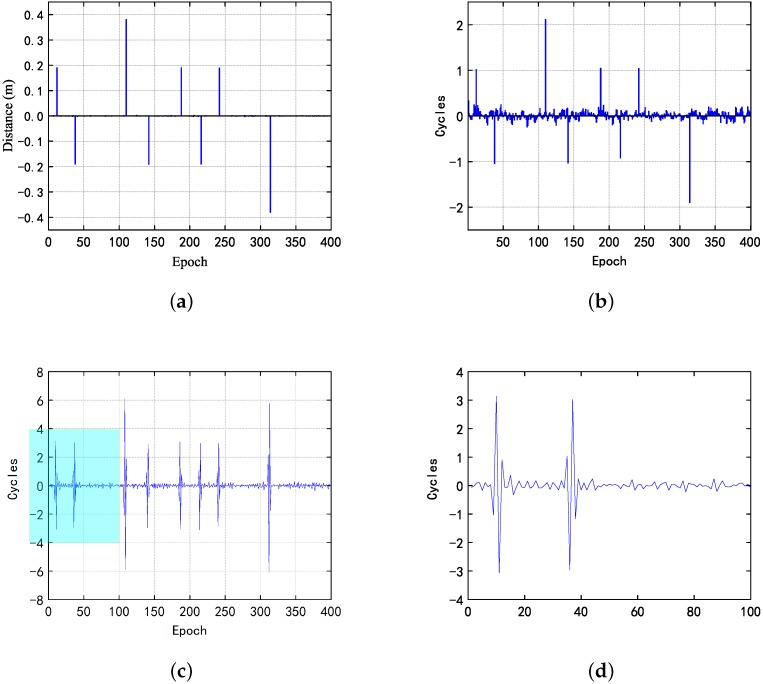
In the first case, (**a**) the results obtained by the proposed indoor dual-frequency cycle slip detection method; (**b**) Doppler method; (**c**) high-order difference method; and (**d**) its details of enlarged local value.

**Figure 7:**
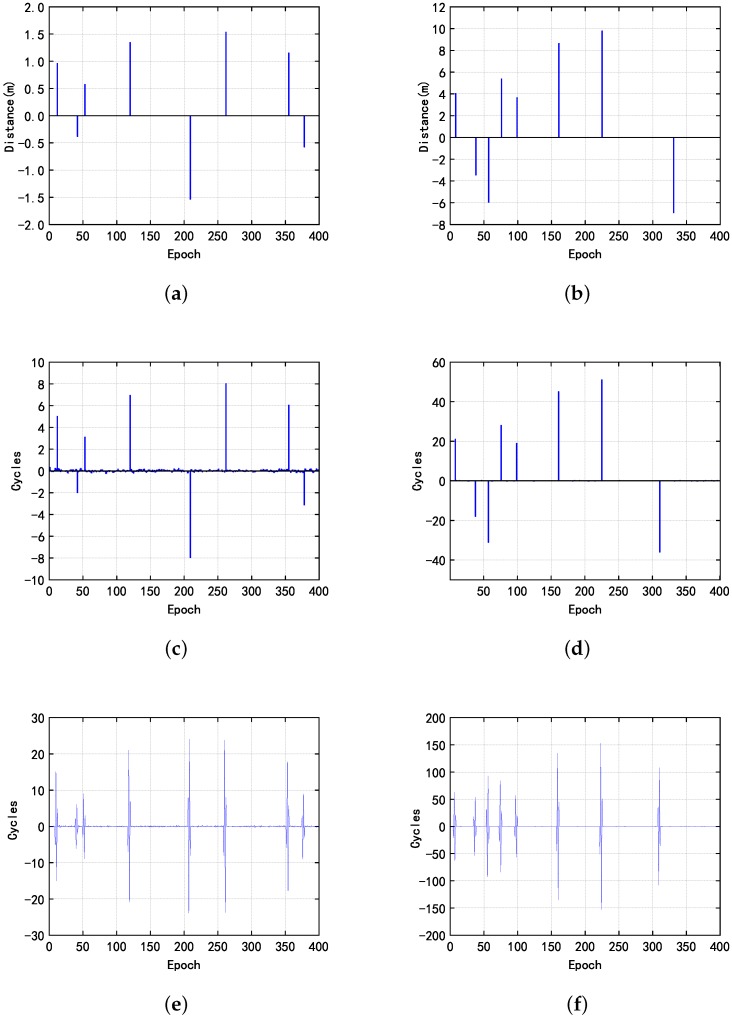
The second case, the three columns on the left, the results obtained by three different methods, (**a**) proposed indoor dual-frequency cycle slip detection method; (**b**) the proposed indoor dual-frequency cycle slip detection method; (**c**) Doppler method; (**d**) Doppler method; (**e**) high-order difference method in the third case, the three columns on the right, the results obtained by three different methods; (**f**) high-order difference method.

As can be seen from the Figure 6, in the first case, one cycle slip occurred on the 12, 38, 142, 188, 216, and 242 epoch, and two cycle slips occurred on the 110, 314 epoch. Also, the proposed method is less affected by noise than the other two methods. The details of the high-order difference method have been enlarged and displayed in the image. Due to the multiple subtractions, there are four peaks in each occurrence of the cycle jump, and the two peaks in the middle are the result of triple the value of the cycle jump, which is beneficial for detecting a small cycle slip. However, the noise error has been amplified at the same time. In order to investigate if the three methods gave the same trend, the data was plotted in Figure 7.

**Figure 8:**
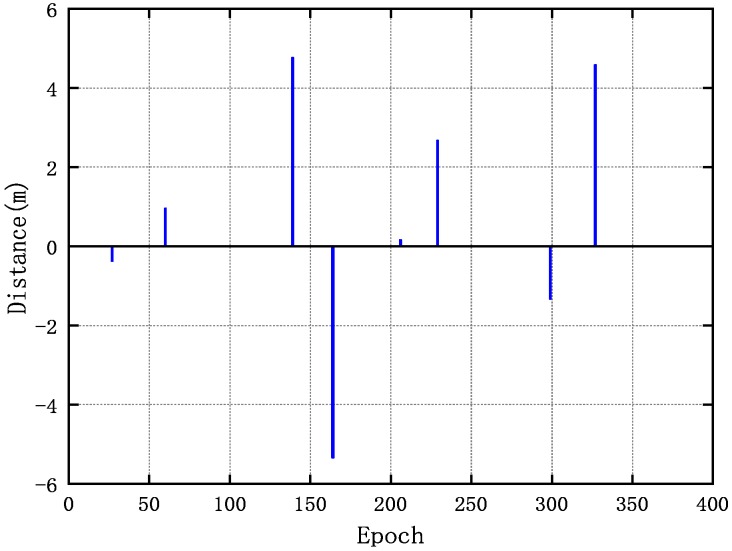
The fourth case, when the cycle slip occurs simultaneously on two frequencies, the results obtained by the proposed indoor dual-frequency cycle slip detection method.

Since the value of the cycle slip is much larger than the first case, the effect of noise on cycle slip detection is small. In the second and third cases, all three methods have a good performance. In order to identify whether it is possible to detect and repair the cycle slip of two frequencies at the same time, we tested the proposed method for the fourth case as shown in the Figure 8.

**Figure 9:**
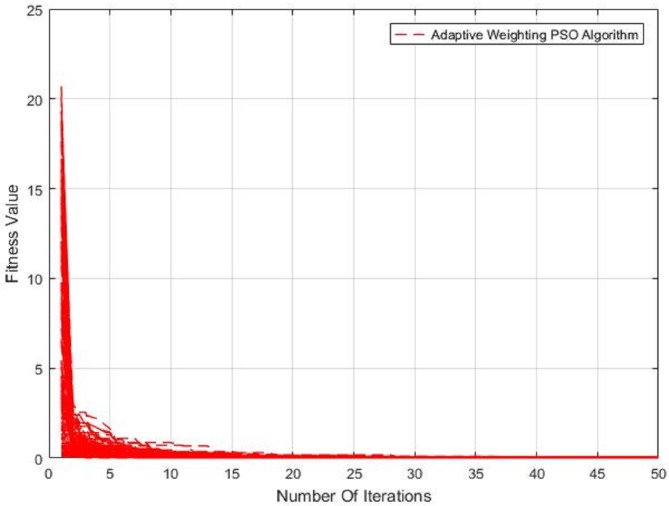
Convergence curve of PSO algorithm.

The results of the proposed method need to be processed by the PSO algorithm when it is used to repair the carrier phase. In order to verify the efficiency of the PSO algorithm, the algorithm has been processed hundreds of times, as seen in Figure 9.

We set the number of particle populations to 30 and the number of iterations to 50. As can be seen from Figure 9, in the hundreds of cycle calculations, the minimum value of fitness can be found before the tenth iteration. The average number of iterations convergence to the optimal value is 8.6, regardless of the size of the cycle slips (one or two frequencies occur cycle slips at the same time). The proposed new cycle slip detection algorithm requires at around 300 calculation of fitness to find the result. However, if the traditional traversal method is used without the help of pseudo-range information, assuming that the size of the cycle slips occurred on B1 is *m* and L1 is *n*, at least m∗n calculation of fitness is required. Usually, in order to make the results more reliable, the size of the search range is often several times larger than *m*, *n*. Assuming that *m* and *n* are both 50, and the expanded search range is two times, the required calculation amount is 10,000, which is much larger than the algorithm we proposed.

**Figure 10:**
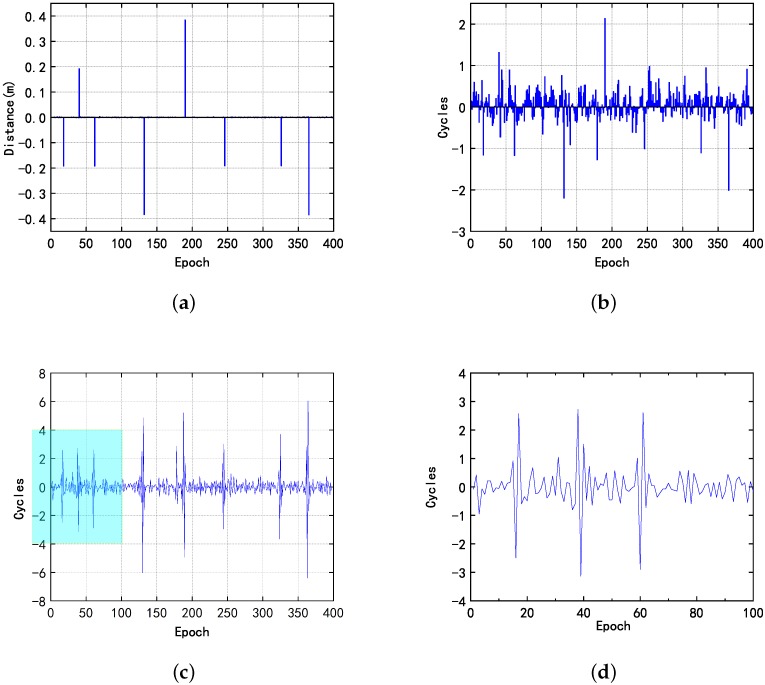
The first case, (**a**) the results obtained by the proposed indoor dual-frequency cycle slip detection method; (**b**) Doppler method; (**c**) high-order difference method; and (**d**) its details of enlarged local value.

**Figure 11:**
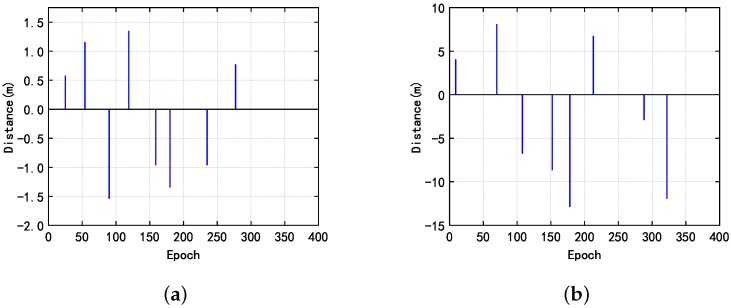

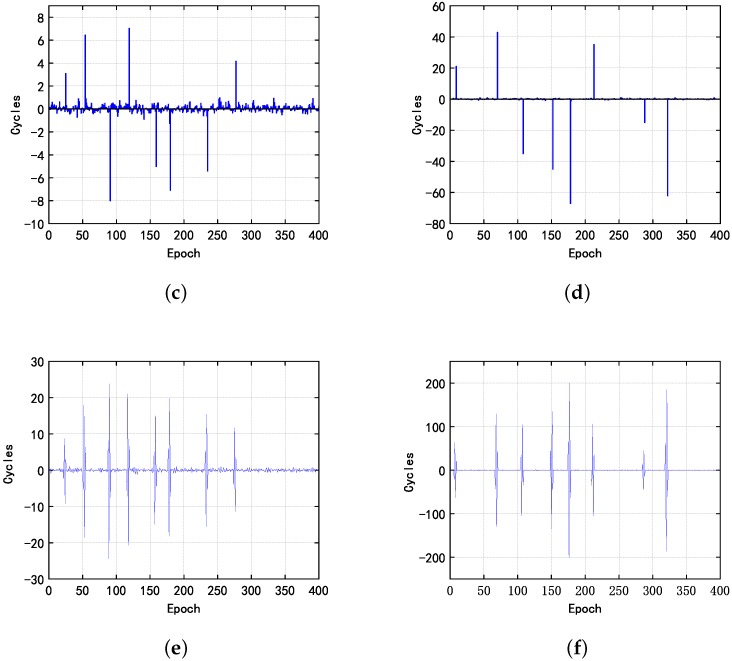
In the second case, the three columns on the left, the results obtained by three different methods (**a**) proposed indoor dual-frequency cycle slip detection method; (**b**) the proposed indoor dual-frequency cycle slip detection method; (**c**) Doppler method; (**d**) Doppler method; (**e**) high-order difference method in the third case, the three columns on the right, the results obtained by three different methods; (**f**) high-order difference method.

**Figure 12:**
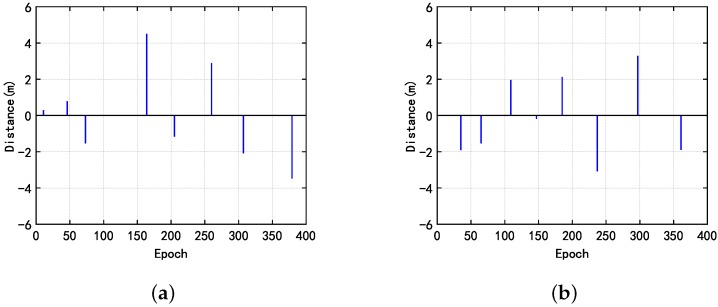

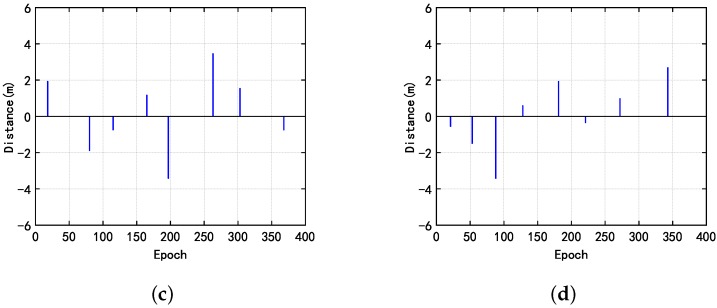
In the fourth case, the results at different sampling rates. (**a**) sampling period is 2 s; (**b**) sampling period is 3 s; (**c**) sampling period is 4 s; (**d**) sampling period is 5 s.

In order to verify the performance of the algorithm under lower sampling rates condition, we processed the phase observation at a rate of 2 s, 3 s, 4 s, 5 s by using the three methods. The test results under different cases at a rate of 3 s, are shown in Figure 10 and Figure 11. The results detected and corrected the cycle slip in case 4 at a rate of 2 s, 3 s, 4 s, 5 s, as shown in Figure 12.

**Figure 13:**
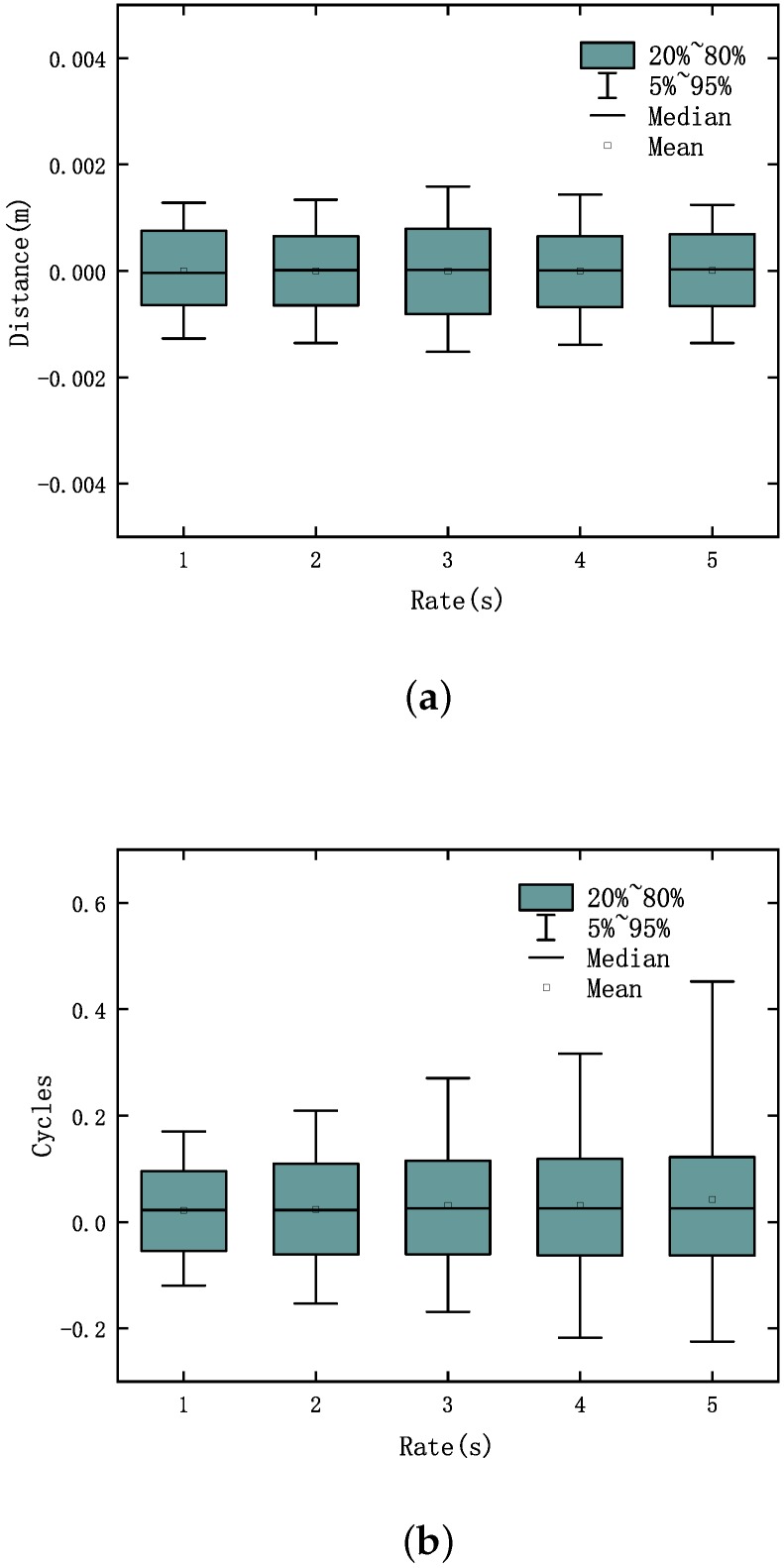

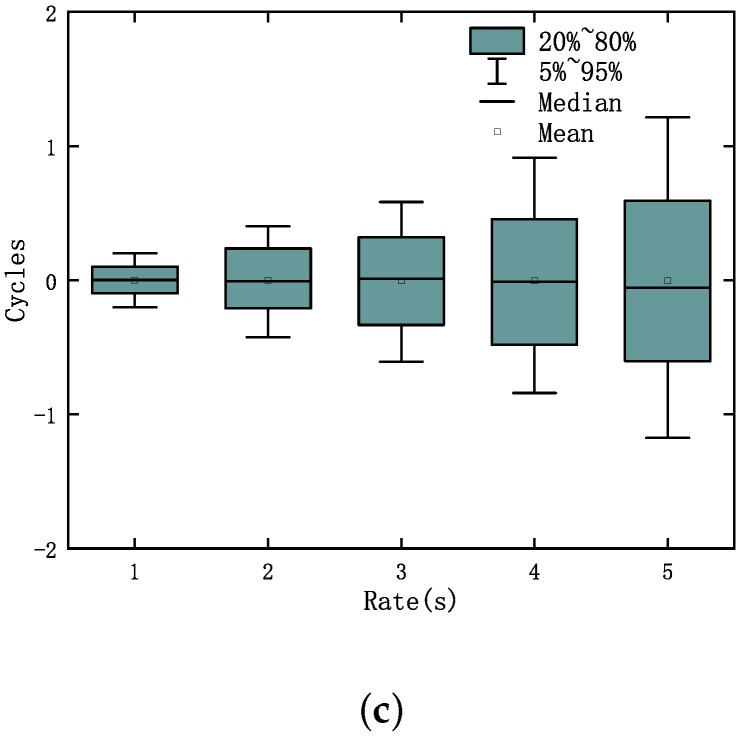
The Box-plot at different sampling rates: (**a**) dual-frequency cycle slip detection method; (**b**) Doppler method; and (**c**) high-order difference method.

When the sampling rate is lower, the noise of the latter two methods is much larger than the proposed method. In order to describe their changes in sampling rate trends more clearly, we have drawn their Box-plot separately as shown in Figure 13. Our proposed method remains accurate and with low noise with decreasing sampling frequency. In the other two methods, with the decrease of the sampling rate, the performance significantly reduced, and the noise range expanded, which affected cycle slip detection in the first case and affected the accuracy of the repair in all cases.

The percentages of detection using the proposed method are at 99%. We compared the correction results of the algorithm with the number of cycle slips added and find the percentages. This method is a statistical result. The percentages of repaired cycle slips are maintained at around 95% regardless of the sampling rate. The repaired percentages of the latter two methods drop sharply as the sampling rate decreases (as shown in Table 3, Table 4, Table 5 and Table 6). Our proposed method has better robustness than the other two cases at lower sampling frequencies. However, it is difficult to detect when the two frequencies have one cycle slip at the same time since the two frequencies are very close. Fortunately, their sizes are often different during actual cycle jumps. Two closer frequencies make the repair of cycle slips difficult. When the signal quality is poor and the noise is greater, the percentages of repaired cycle slips will decrease.

## 5. Discussion and Conclusions

There are many methods to detect and repair cycle slips. However, it is still a challenging issue, in particular, in the case of an indoor environment. Our research aimed to examine the results of the dual-frequency combination of B1 and L1 for cycle slip detection in the indoor multipath environment and investigate the performance of the adaptive weight PSO algorithm for a cycle slip repair. In order to test the proposed method comprehensively, we set four different cases and compared them with the traditional cycle slip detection method by using real statistical pseudolite data. One advantage of the method is that it avoids the problem of the ionosphere effect. These experiments confirmed that the new method is not affected by the sampling rate, and the percentages of detection can maintain more than 99%. The findings of this study suggest that the combination of B1 L1 frequency can also be used for dual-frequency cycle slip detection instead of B1, B2, or L1, L2. The proposed method has more efficiency in cycle slip repair by using the adaptive weight PSO algorithm compared to the traditional method. The question raised by this study is whether the cycle slip detection and repair method improves the accuracy of indoor positioning. In addition, our data is collected in an ideal environment. More situations, experiments with larger sample data need to be performed. The results will be improved if a smoother filter is used to reduce noise. Further research should be undertaken to explore the improvement of robustness for positioning in an indoor multipath environment.

## Figures and Tables

**Table 1:** Coordinates of transmitter antennae.

Station	X (m)	Y (m)	Z (m)
S1	0	0	3.000
S2	−2.378	−3.677	3.105
S3	0.041	−3.396	2.981
S4	−2.816	−2.082	2.946
S5	−1.416	−0.871	3.177
S6	−2.791	−0.816	3.122
S7	−1.331	−2.171	3.117

**Table 2:** The maximum and minimum SNR (signal-to-noise ratio) values and variances.

Pseudolite Number	B1 SNR (dB)			L1 SNR (dB)		
	MAX	MIN	STD	MAX	MIN	STD
S1	48	46	0.40	48	46	0.35
S2	47	45	0.39	49	48	0.33
S3	52	52	0	49	49	0
S4	52	51	0.09	49	48	0.24
S5	52	51	0.04	49	49	0
S6	52	52	0	49	48	0.14
S7	46	43	0.62	41	37	0.53

**Table 3:** The results of detected and corrected of the cycle slip over all stations for the three different methods in case 1.

Rate (s)	Detection (%)			Repair (%)		
	I	II	III	I	II	III
1	100	99	98	96	98	97
2	100	98	97	94	91	94
3	100	94	95	95	85	87
4	100	93	93	94	82	77
5	100	91	92	96	77	72

**Table 4:** The results of detected and corrected of the cycle slip over all stations for the three different methods in case 2.

Rate (s)	Detection (%)			Repair (%)		
	I	II	III	I	II	III
1	100	100	100	96	97	96
2	100	100	100	95	92	95
3	100	100	99	95	84	88
4	100	100	98	96	81	75
5	100	99	98	94	76	71

**Table 5:** The results of detected and corrected of the cycle slip over all stations for the three different methods in case 3.

Rate (s)	Detection (%)			Repair (%)		
	I	II	III	I	II	III
1	100	100	100	95	97	96
2	100	100	100	95	93	93
3	100	100	100	96	88	85
4	100	100	100	95	83	73
5	100	100	100	94	79	68

**Table 6:** The results of detected and corrected of the cycle slips in case 4.

Sampling Period	1 (s)	2 (s)	3 (s)	4 (s)	5 (s)
Detection (%)	100	100	99	100	99
Repair (%)	95	94	93	94	94
